# Massive hemoptysis treated with embolization of an ectopic bronchial artery arising from the right thyrocervical trunk: a case report

**DOI:** 10.1186/s42155-022-00285-3

**Published:** 2022-01-18

**Authors:** Songhyon Cho, Kenji Kubota, Yoshikazu Hirose, Norihiko Yoshimura, Yui Murai, Yasuo Hirose

**Affiliations:** 1 Department of Emergency and Critical Care Medical Center, Niigata City General Hospital, 463-7, Shumoku, Niigata, 950-1197 Japan; 2 Department of Radiology, Niigata City General Hospital, 463-7, Shumoku, Niigata, 950-1197 Japan; 3 Department of Respiratory Medicine, Niigata City General Hospital, 463-7, Shumoku, Niigata, 950-1197 Japan

**Keywords:** Hemoptysis, Embolization, Bronchial artery, Multidetector computed tomography, Computed tomography angiography

## Abstract

**Background:**

Ectopic bronchial artery and non-bronchial systemic arteries may be the culprit vessels of hemoptysis. The main cause of clinical failure of bronchial artery embolization is incomplete embolization caused by the misidentification of the culprit arteries by conventional angiography. Multidetector computed tomography angiography is useful for visualizing the culprit arteries.

**Case presentation:**

An 82-year-old man was admitted with hemoptysis. Preprocedural multidetector computed tomography angiography revealed an ectopic bronchial artery branching from the right thyrocervical trunk. Superselective embolization of the ectopic bronchial artery was performed using gelatin sponge particles and metallic coils. Hemoptysis was controlled by this procedure without any associated complications.

**Conclusions:**

Ectopic bronchial arteries originating from the thyrocervical trunk are rare. Preprocedural multidetector computed tomography angiography is useful for visualizing the culprit arteries of hemoptysis, especially if a patient has an ectopic bronchial artery or an ectopic non-bronchial systemic artery.

## Introduction

Massive hemoptysis is one of the fatal respiratory symptoms and is most frequently bronchogenic. Bronchial artery embolization (BAE) is widely used to manage massive hemoptysis (Yoon et al. 2002). However, ectopic bronchial artery (BA) and non-bronchial systemic arteries (NBSAs), such as the subclavian, internal mammary, or inferior phrenic arteries, may be the culprit vessels of hemoptysis. The main cause of clinical failure of BAE is incomplete embolization caused by the misidentification of the culprit arteries by conventional angiography (Zhao et al. 2017). Multidetector computed tomography (MDCT) angiography is useful for visualizing the ectopic origin of the BA and NBSAs during BAE that can be easily missed by conventional angiography (Li et al. 2019). We report a rare case of an ectopic BA that originated from the right thyrocervical trunk. It was detected by preprocedural MDCT angiography and was completely embolized to control massive hemoptysis.

## Case presentation

An 82-year-old man with hemoptysis was admitted to our hospital. He had a history of bronchiectasis, but this was the first time he had ever developed hemoptysis. He had first gone to a neighboring hospital and received antibiotics intravenously. He was referred to our hospital for further evaluation and management. Despite conservative management, including aggressive antimicrobial treatment, the patient experienced massive hemoptysis on the third day after admission and underwent emergency BAE.

**Fig. 1:**
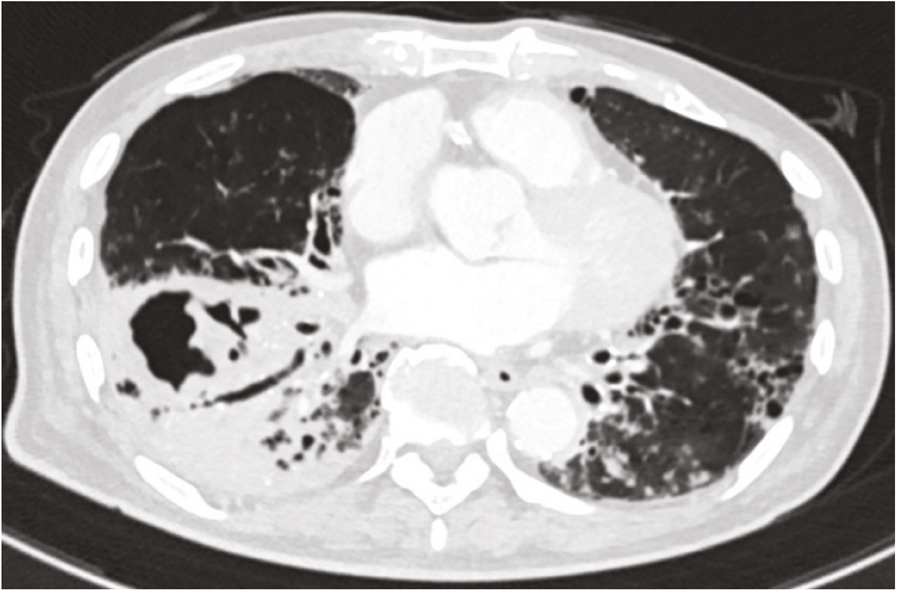
The chest CT lung window imaging shows bilateral bronchiectasis and a large bulla with surrounding consolidation in the right lower lobe

**Fig. 2:**
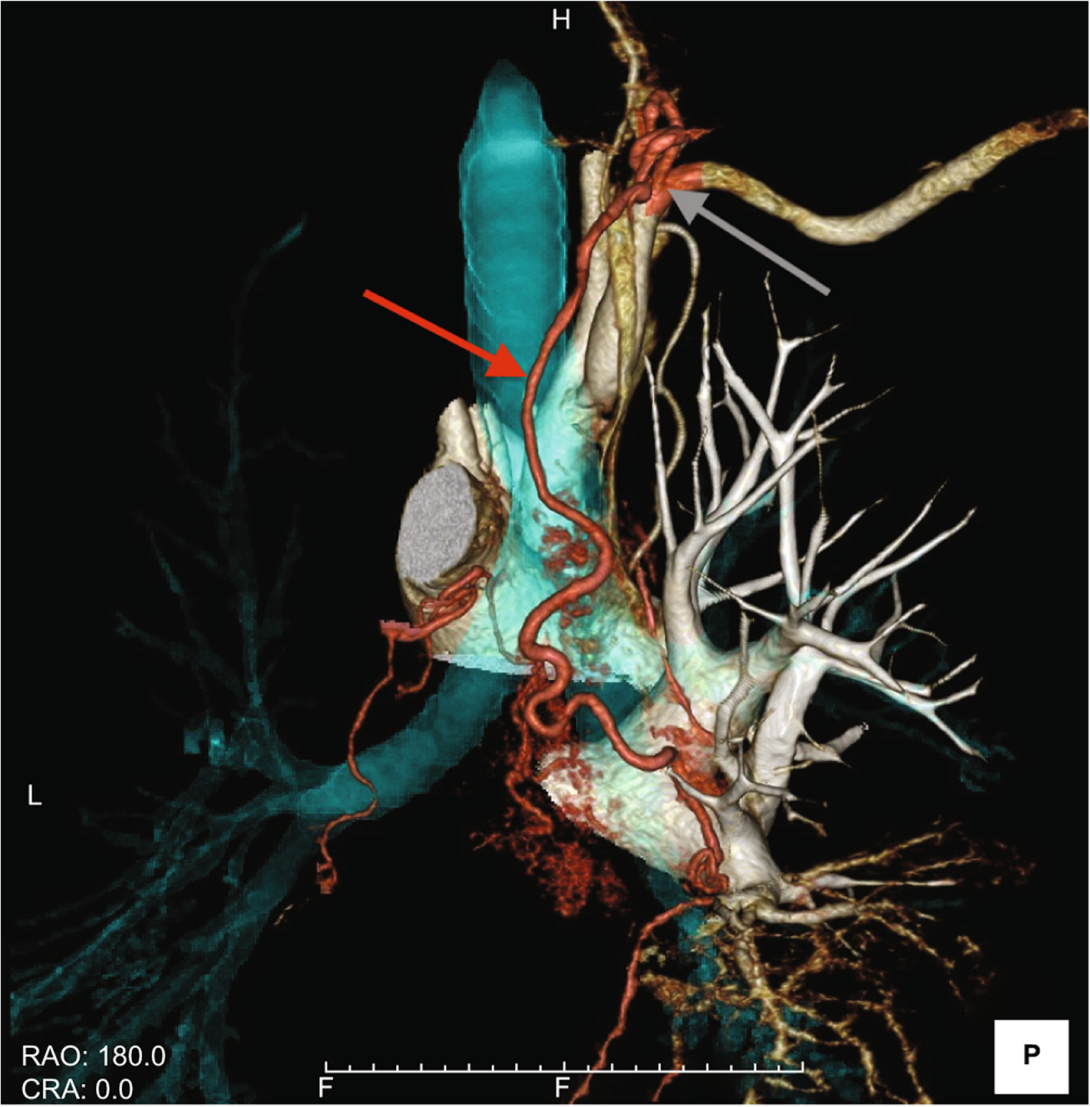
Volume rendering reconstructed image of multidetector computed tomography angiography shows a hypertrophic ectopic right bronchial artery (red arrow) arising from the right thyrocervical trunk (gray arrow)

**Fig. 3:**
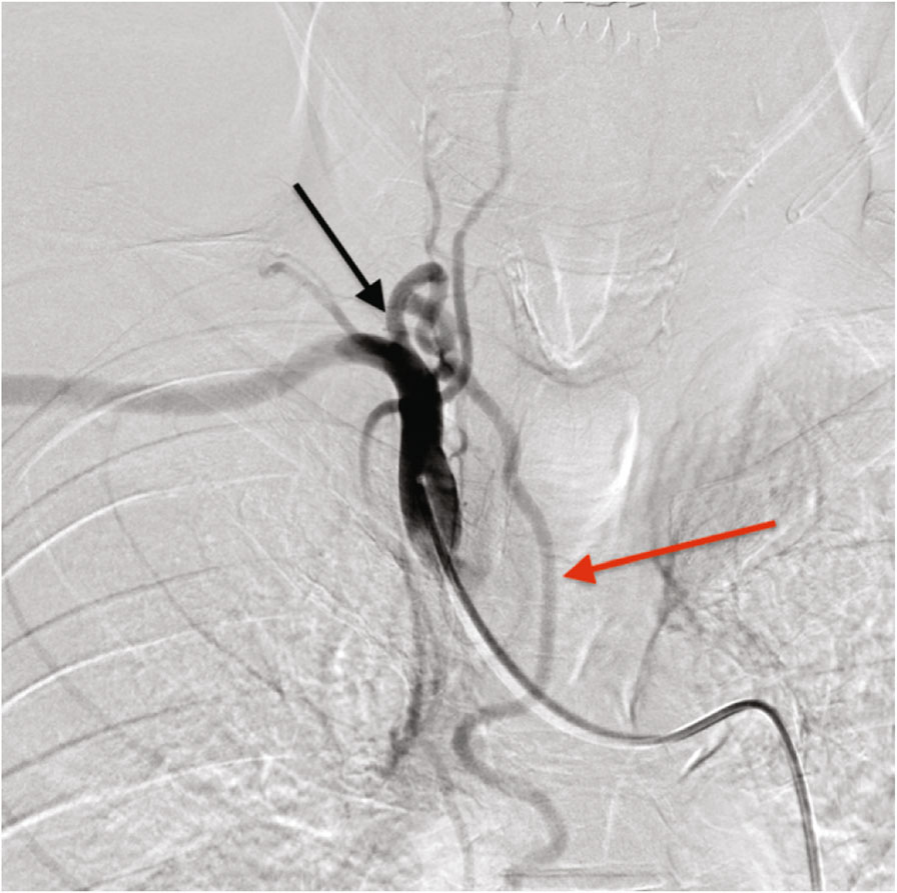
Angiogram of the brachiocephalic artery shows the hypertrophic ectopic bronchial artery (red arrow) arising from the right thyrocervical trunk (black arrow), findings that corresponded with the CTA image

Before emergency BAE, he underwent preprocedural evaluation by contrast-enhanced computed tomography (CT) scan with a 128-slice scanner (Siemens SOMATOM Drive, Siemens Healthineers, Tokyo, Japan), with the arterial phase 30 s after, and the delayed phase 90 s after intravenous contrast administration (100 mL Oypalomin 370 mg/mL, Fuji Pharma, Tokyo, Japan) at 3 mL/s. The chest CT lung window imaging showed bilateral bronchiectasis and a large bulla with surrounding consolidation in the right lower lobe, which was thought to be the source of hemoptysis (Fig. 1). Furthermore, computed tomography angiography revealed an ectopic BA that arose from the right thyrocervical trunk, supplying the right lower lobe in addition to the normal right BA (Fig. 2). This ectopic BA was significantly hypertrophied compared to the normal right BA, suggesting that the ectopic BA was the culprit vessel of the hemoptysis (Fig. 3).

**Fig. 4:**
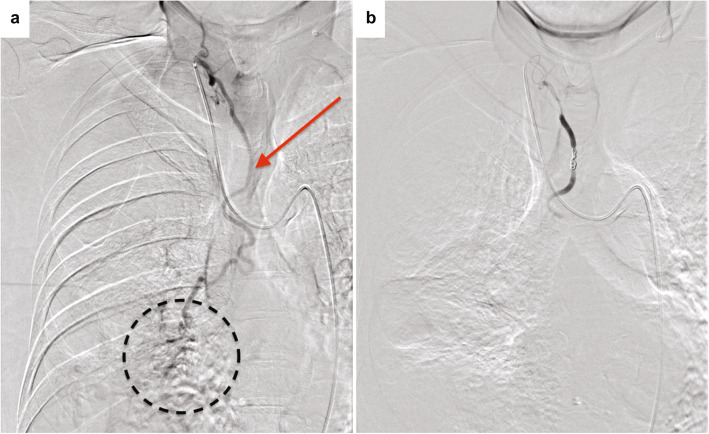
**A** Selective angiogram of the ectopic right bronchial artery (arrow) shows parenchymal staining in the right lower lobe (dotted circle). **B** The parenchymal staining disappeared after embolization with gelatin sponge particles

**Fig. 5:**
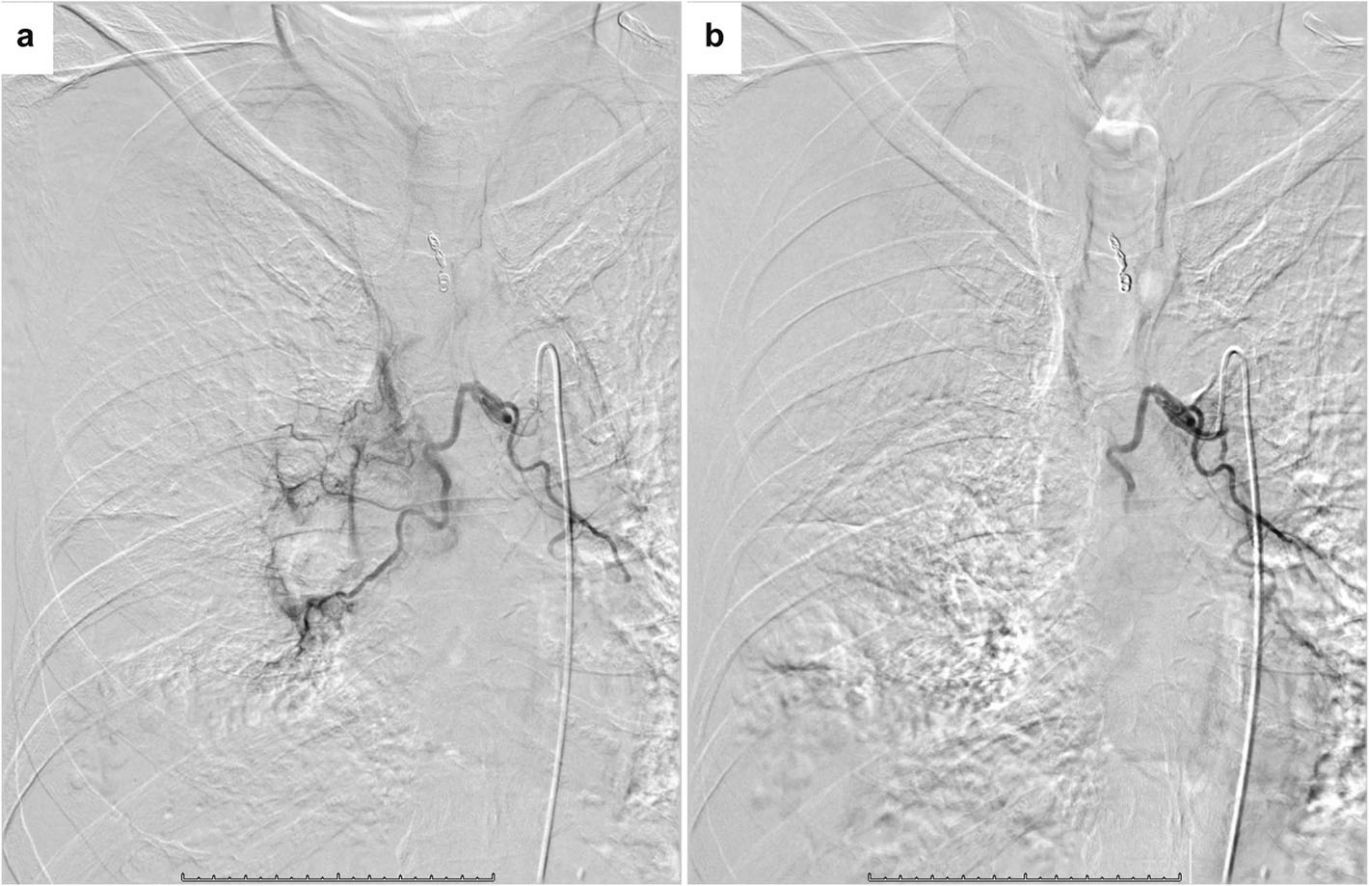
**A** Selective angiogram of the common bronchial trunk shows normal bilateral bronchial arteries. **B** Only right BA was selectively embolized with gelatin sponge particles

Superselective embolization of the ectopic BA was performed with gelatin sponge particles and metallic coils (Tornado, Cook Medical, Bloomington, USA) with a 1.9–2.9 Fr microcatheter (Breakthrough, Boston Scientific Japan, Tokyo, Japan) (Fig. 4A, B). After superselective embolization of the ectopic BA, the normal right BA was selectively embolized with gelatin sponge particles (Fig. 5A, B).

Hemoptysis was controlled by this procedure without any procedural complications. He was discharged on day 5 of hospitalization with no complications.

## Discussion

The BAs are the source of massive hemoptysis in > 90% of cases (Lorenz et al. 2012). Since the report published in 1973 by Remy et al., BAE has been revealed as an effective technique for the control of massive hemoptysis. However, interventional radiologists who perform BAE should keep in mind that the BA may show anatomical variations in terms of origin, branching pattern, and course (Yoon et al. 2002). Furthermore, a minority of massive hemoptysis result from NBSAs or pulmonary arteries (Lorenz et al. 2012).

BAs originating apart from between the T5 and T6 vertebrae are considered ectopic, and the incidence of ectopicity has been reported to range from 8.3% to 36% (Michimoto et al. 2020). Ectopic BAs can be distinguished from NBSAs by their course along the major bronchi. On the other hand, NBSAs enter the pulmonary parenchyma through the adherent pleura or by way of the pulmonary ligament, and their course is not parallel to that of the bronchi (Sancho et al. 1998). In the present case, the branches of the right thyrocervical trunk ran along the main bronchus; therefore, this vessel was determined to be an ectopic BA rather than an NBSA.

Ectopic BAs originating from the thyrocervical trunk are rare. Summarizing several studies on MDCT angiography (Michimoto et al. 2020; Hartmann et al. 2007; Battal et al. 2011; Yener et al. 2015), only 9 of 624 patients (1.4%) had BAs originating from the thyrocervical trunk. This rate was lower than those of the subclavian (29/624; 4.6%) or internal mammary (11/624; 1.8%) artery origin. To our knowledge, most of the candidates for BAE for hemoptysis from a BA originating from the thyrocervical trunk have been reported to have a history of cystic fibrosis (Yoon et al. 2002; Hartmann et al. 2007; Battal et al. 2011; Yener et al. 2015). Furthermore, BAE was performed several times in these cases. In contrast, the patient in the present case had no history of cystic fibrosis and underwent BAE for the first time.

The rate of hemoptysis recurrence after BAE ranges from 9%–55% (Michimoto et al. 2020), and Zhao et al. reported that the main cause of clinical failure of BAE is incomplete embolization caused by misidentification of the culprit arteries by conventional angiography, especially for ectopic BAs and NBSAs (Zhao et al. 2017). A systematic review by Panda et al. (2017) reported that inadequate technique or incomplete embolization due to failure to detect all culprit arteries leads to early recurrence of hemoptysis within 3 months of BAE.

Many studies have reported that MDCT angiography is not only able to identify the source of bleeding and the underlying disease of hemoptysis, but also precisely detect the origins and courses of culprit arteries before BAE, which is especially advantageous for visualizing the ectopic origin of BAs and NBSAs, which are easily missed by conventional angiography during BAE (Zhao et al. 2017; Li et al. 2019; Lorenz et al. 2012; Michimoto et al. 2020; Panda et al. 2017). Li et al. (2019) suggested that preprocedural MDCT angiography can reduce the recurrence rate of hemoptysis after BAE, and recommended MDCT angiography as a routine examination before BAE in patients with hemoptysis as far as possible. In the present case, visualization of the ectopic BA from the right thyrocervical trunk on preprocedural MDCT allowed us to perform a successful BAE. We recommend that preprocedural MDCT be performed so that the thyrocervical trunk can also be evaluated. In this case, the region proximal to the ectopic BA was occluded, the patient was discharged, and no rebleeding was observed. However, Yoon et al. (2002) argue that a coil should not be used for BAE because re-embolization is precluded if hemoptysis recurs. Therefore, there was an option to not use proximal coiling of the ectopic BA.

## Conclusion

We performed BAE in a patient with an ectopic BA of the right thyrocervical trunk. Preprocedural MDCT angiography is useful for visualizing the culprit arteries of hemoptysis, especially if a patient has an ectopic origin of BAs or NBSAs.

## Data Availability

Not applicable.

## Abbreviations

BAE: Bronchial artery embolization
BA: Bronchial artery
NBSAs: Non-bronchial systemic arteries
MDCT: Multi-detector computed tomography
CT: computed tomography
